# Single-nucleotide and long-patch base excision repair of DNA damage in plants

**DOI:** 10.1111/j.1365-313X.2009.03994.x

**Published:** 2009-09-01

**Authors:** Dolores Córdoba-Cañero, Teresa Morales-Ruiz, Teresa Roldán-Arjona, Rafael R Ariza

**Affiliations:** Department of Genetics, University of Córdoba14071 Córdoba, Spain

**Keywords:** abasic sites, Arabidopsis, DNA polymerase, DNA repair, uracil

## Abstract

Base excision repair (BER) is a critical pathway in cellular defense against endogenous or exogenous DNA damage. This elaborate multistep process is initiated by DNA glycosylases that excise the damaged base, and continues through the concerted action of additional proteins that finally restore DNA to the unmodified state. BER has been subject to detailed biochemical analysis in bacteria, yeast and animals, mainly through *in vitro* reproduction of the entire repair reaction in cell-free extracts. However, an understanding of this repair pathway in plants has consistently lagged behind. We report the extension of BER biochemical analysis to plants, using Arabidopsis cell extracts to monitor repair of DNA base damage *in vitro*. We have used this system to demonstrate that Arabidopsis cell extracts contain the enzymatic machinery required to completely repair ubiquitous DNA lesions, such as uracil and abasic (AP) sites. Our results reveal that AP sites generated after uracil excision are processed both by AP endonucleases and AP lyases, generating either 5′- or 3′-blocked ends, respectively. We have also found that gap filling and ligation may proceed either through insertion of just one nucleotide (short-patch BER) or several nucleotides (long-patch BER). This experimental system should prove useful in the biochemical and genetic dissection of BER in plants, and contribute to provide a broader picture of the evolution and biological relevance of DNA repair pathways.

## Introduction

Maintaining genome integrity in the face of constant DNA damage induced by spontaneous chemical changes or genotoxic agents is an essential task for all organisms (Friedberg *et al.*, 2006; Lindahl, 1993). A major pathway for protecting DNA from endogenous or exogenous damage is base excision repair (BER). This is a multistep process initiated by DNA glycosylases that excise damaged or incorrect bases and generate an abasic (apurinic/apyrimidinic, AP) site (Stivers and Jiang, 2003; Zharkov and Grollman, 2005). AP sites may also arise from normal bases by spontaneous hydrolysis of the *N*-glycosylic bond (Lindahl, 1993). The subsequent steps required to complete the repair include incision at the AP site, generation of a gap, repair synthesis and ligation (Fortini and Dogliotti, 2007).

BER has been extensively studied in higher eukaryotes. In mammalian cells, AP sites generated during BER are usually processed by AP endonucleases that create an incision in the 5′ side of the AP site, leaving 3′-OH and 5′-deoxyribose-5-phosphate (5′-dRP) termini (Dianov *et al.*, 1992; Levin and Demple, 1990). The ensuing gap filling may take place either by insertion of a single nucleotide (short-patch repair, SP) or by DNA synthesis involving several nucleotides (long-patch repair, LP) (Fortini and Dogliotti, 2007). In the SP BER subpathway, gap filling is performed by DNA polymerase β (Pol β), which is also endowed with dRPase activity that releases the blocking 5′-dRP end and allows DNA ligation (Srivastava *et al.*, 1998). In the LP BER subpathway, DNA synthesis may be performed by replicative DNA polymerases δ or ε, which carry out displacement of the strand containing the 5′-dRP terminus, and generate a ‘flap’ structure that is excised by the 5′-flap endonuclease FEN1 before ligation (Pascucci *et al.*, 1999). Both SP and LP BER have been reconstituted *in vitro* using purified proteins (Dianov and Lindahl, 1994; Klungland and Lindahl, 1997; Kubota *et al.*, 1996; Nicholl *et al.*, 1997; Pascucci *et al.*, 1999; Srivastava *et al.*, 1998), and the choice between the subpathways in cells may depend on various factors, such as the nature of the lesion and the type of DNA glycosylase that initiates the BER process (Fortini and Dogliotti, 2007).

As experimental data accumulate for non-mammalian systems, it is becoming clear that there are significant differences in the strategies employed by different species during the post-excision events that take place in BER (Kelley *et al.*, 2003). Therefore, a complete understanding of this repair pathway will require mechanistic studies involving species from all main groups of organisms. However, plants have usually been neglected in the biochemical and genetic analysis of DNA repair pathways, which have generally focused on bacterial, yeast and mammalian systems (Friedberg *et al.*, 2006). Recent research efforts are rapidly changing this, and substantial knowledge has begun to accumulate about plant DNA repair processes (Bray and West, 2005; Britt, 2002; Hays, 2002; Kunz *et al.*, 2005). Numerous studies mostly performed in Arabidopsis have shown that plants are equipped with structural and/or functional homologs of most of the BER proteins identified in other organisms (Britt, 2002; Hays, 2002; Roldan-Arjona and Ariza, 2009). Additionally, recent genetic and biochemical evidence has revealed that plant BER performs a key role in epigenetic regulation, through active DNA demethylation initiated by 5-methylcytosine DNA glycosylases (Agius *et al.*, 2006; Gehring *et al.*, 2006; Morales-Ruiz *et al.*, 2006). Therefore, unraveling the enzymatic mechanisms of BER will be critical to understand its full relevance for plant physiology.

Much of the knowledge gathered during the past 20 years about the enzymology of animal and microbial BER has been obtained through the use of cell-free *in vitro* assays that allow the steps of the repair process to be monitored (for a review, see Dianov, 2003). The complete BER pathway has been reproduced *in vitro* using extracts from *Escherichia coli* (Dianov *et al.*, 1992), *Saccharomyces cerevisiae* (Wang *et al.*, 1993), *Schizosaccharomyces pombe* (Alseth *et al.*, 2004), *Xenopus laevis* (Matsumoto and Bogenhagen, 1989, 1991), *Plasmodium falciparum* (Haltiwanger *et al.*, 2000), *Caenorhabditis elegans* (Shatilla and Ramotar, 2002) and human cells (Dianov *et al.*, 1992). However, to date, there are no reports describing assays for specifically detecting BER in plant cells. This has been a persistent obstacle to understanding the relevance of this DNA repair pathway in plants, leaving many significant questions unanswered. In this study, we have extended the biochemical analysis of BER to plants by developing a specific assay to monitor the repair of damaged bases in Arabidopsis whole-cell extracts. Using this assay we have found that repair intermediates arising during plant BER may be processed both by AP lyases and AP endonucleases. We have also established that uracil and AP site repair may be completed both via single-nucleotide insertion and by long-patch DNA synthesis. We have also explored the DNA polymerase requirements for the gap-filling step. We discuss our findings in the light of what is currently known about plant DNA polymerases.

## Results

### Uracil excision repair in Arabidopsis whole-cell extracts

**Figure 1:**
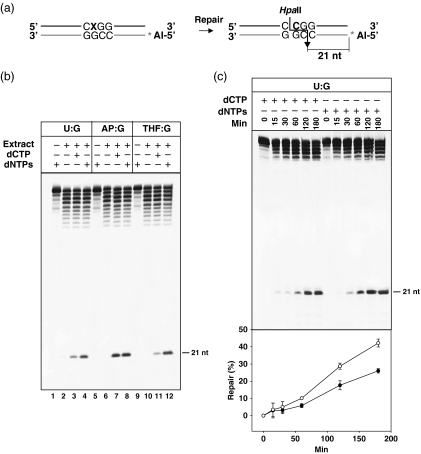
Repair of base DNA damage by Arabidopsis cell extracts. (a) Schematic diagram of molecules used as DNA substrates. Double-stranded oligonucleotides contained a lesion (X = uracil or AP site) at an *Hpa*II site on the upper strand. The Alexa Fluor-labeled 5′ end of the lower strand is indicated by a star. The size of the 5′ end-labeled fragment generated after *Hpa*II digestion of the fully repaired product is indicated on the right. (b) DNA duplexes containing U, a natural AP site, or THF opposite G were incubated with Arabidopsis cell extract at 30°C for 3 h in a reaction mixture containing either dCTP or all four dNTPs, as indicated. Reaction products were digested with *Hpa*II, separated in a 12% denaturing polyacrylamide gel and quantified by fluorescence scanning. (c) A DNA duplex containing a U:G mispair was incubated with Arabidopsis cell extracts at 30°C in a reaction mixture containing either dCTP or all four dNTPs. Reactions were stopped at the indicated times and products were analyzed as described above. The percentage of fully repaired DNA product for reactions containing only dCTP (•) or all four dNTPs (○) is shown in the bottom panel. Values are means with standard errors from two independent experiments.

We chose uracil as a convenient model lesion to investigate BER in Arabidopsis cells, as uracil-initiated BER is well documented in different prokaryotic and eukaryotic systems (Friedberg *et al.*, 2006). Uracil in DNA may arise either from spontaneous deamination of cytosine or from erroneous incorporation of dUMP instead of dTMP, leading to mutagenic U:G mispairs or to U:A pairs, respectively (Kavli *et al.*, 2007). To establish an *in vitro* system for BER, we used a 51-mer duplex oligonucleotide labeled at the 5′ end of the lower strand, with a single U residue within an *Hpa*II recognition site on the upper strand, as the DNA substrate (Figure 1a). The presence of U instead of C at the restriction site makes the DNA substrate refractory to *Hpa*II cleavage. In this system, uracil repair is detected as a conversion of duplex DNA to a form susceptible to *Hpa*II digestion, visualized as a 21-nt-labeled fragment following polyacrylamide gel electrophoresis (Figure 1a).

Extracts obtained from snap-frozen 15-day-old seedlings (see Experimental procedures) efficiently and reproducibly converted the uracil-containing DNA to a form susceptible to *Hpa*II digestion (Figure 1b). Importantly, repair was dependent on the presence of deoxyribonucleotide triphosphates (dNTPs) in the reaction mixture, but was also detected when only deoxycytidine triphosphate (dCTP) was present (Figure 1b, lanes 3 and 4). Equivalent results were observed when the whole-cell extract was incubated with DNA substrates containing either a natural AP site or a synthetic AP site analog (tetrahydrofuran, THF), instead of uracil (Figure 1b, lanes 5–12). Fragments shorter than 51 nt detected in the upper part of the gel are probably the result of exonuclease activity in the extracts, as they were not observed when incubations were performed in the absence of Mg^2+^ (data not shown). We also examined the repair kinetics of a U:G mispair, measuring the level of fully repaired product at different times (Figure 1c). We found that repair was faster when all four deoxynucleotides were present in the reaction mixture, but the rate of repair was also significant when the only deoxynucleotide available was dCTP.

**Figure 2:**
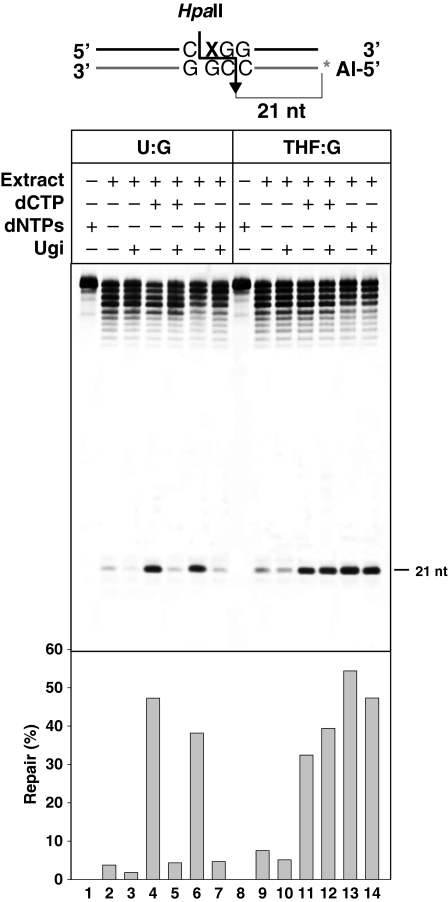
Uracil repair by Arabidopsis cell extracts is dependent on uracil DNA glycosylase (UDG) activity. DNA duplexes containing either U or THF opposite G were incubated with Arabidopsis cell extracts at 30°C for 3 h in a reaction mixture containing either dCTP or all four dNTPs, both in the absence or presence of 2 U of uracil-DNA glycosylase inhibitor (Ugi). Reaction products were digested with *Hpa*II, separated in a 12% denaturing polyacrylamide gel and quantified by fluorescence scanning. The percentage of fully repaired DNA product is shown in the bottom panel.

To assess whether the first step of the repair process is dependent on uracil DNA glycosylase (UDG) activity, we examined the effect of uracil-DNA glycosylase inhibitor (Ugi) on the reaction. This small peptide is a specific inhibitor of UDGs from the UNG family, and has been successfully used to detect UDG-dependent uracil excision in cell extracts from different organisms (Bennett *et al.*, 2001; Shatilla and Ramotar, 2002; Zeitlin *et al.*, 2005). We found that repair of uracil-containing DNA was strongly reduced when Ugi was added to the reaction, in the presence of either dCTP or of all four dNTPs (Figure 2, lanes 5 and 7). In contrast, the addition of Ugi had no significant effect on the repair of an analogous DNA substrate containing a synthetic AP site (Figure 2, lanes 12 and 14). We conclude that Arabidopsis cell extracts are able to fully repair a U:G mismatch through a BER pathway initiated by UDG activity.

### AP sites generated after uracil excision may be processed both by AP endonucleases and AP lyases

**Figure 3:**
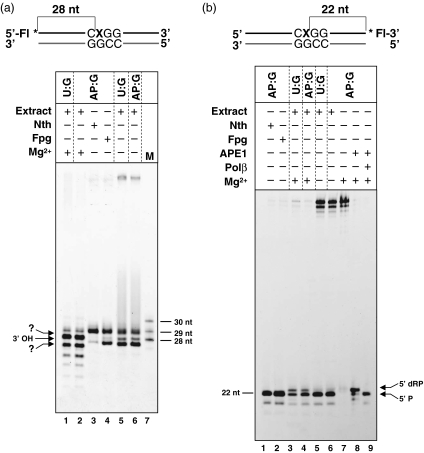
AP sites generated after uracil excision are processed both by AP endonucleases and AP lyases. (a) Analysis of 3′ termini. DNA duplexes containing either U or a natural AP site at the 5′ end-labeled upper strand were incubated at 30°C for 3 h with different combinations of Arabidopsis cell extract, *Escherichia coli* Nth (13 ng), *E. coli* Fpg (20 ng) and MgCl_2_ (5 mm), as indicated. Reaction products were separated in a 12% denaturing polyacrylamide gel, and detected by fluorescence scanning. Size markers corresponding to 28, 29 and 30 nt were loaded on lane 7. Fragments corresponding to β- and δ-elimination products, and to 3′-OH termini are indicated by arrows. (b) Analysis of 5′ termini. DNA duplexes containing either U or a natural AP site at the 3′ end-labeled upper strand were incubated at 30°C for 3 h with different combinations of Arabidopsis cell extract, *E. coli* Nth (13 ng), *E. coli* Fpg (20 ng), human APE1 (10 U), human Pol β (2.4 U) and MgCl_2_ (5 mm), as indicated. Reaction products were stabilized by reduction with NaBH_4_, separated in a 12% denaturing polyacrylamide gel and then detected by fluorescence scanning. Fragments corresponding to 5′-deoxyribose-5-phosphate (5′-dRP) and 5′-phosphate (5′-P) products are indicated by arrows.

We next turned our attention to the nature of the 3′ and 5′ termini generated on the damaged DNA strand following uracil excision. AP sites generated after base excision may be processed by AP endonucleases, but also by the AP lyase activity associated with bifunctional DNA glycosylases/lyases. These two kinds of activities produce very different types of 5′ and 3′ termini (Figure S1). AP endonucleases are usually Mg^2+^-dependent, and they perform hydrolysis at the 5′ side of the AP site, yielding a 3′-OH end and a 5′-dRP terminus (Levin and Demple, 1990). By contrast, AP lyases cleave 3′ to the AP site through a Mg^2+^-independent β-elimination mechanism, leaving a 5′-phosphate (5′-P) end and a 3′-α,β-unsaturated aldehyde (Levin and Demple, 1990); in some cases, they additionally catalyze δ-elimination, generating a 3′-phosphate (3′-P) terminus (Zharkov *et al.*, 2003). Thus, AP endonucleases and AP lyases generate 5′- and 3′-blocked ends, respectively. To determine whether either or both of these two types of termini are generated during repair of uracil or AP sites by Arabidopsis cell-free extracts, we performed repair reactions in the absence or presence of Mg^2+^, using DNA substrates in which the lesion-containing strand was labeled at the 5′ or 3′ end (Figure 3).

We first examined the 3′ terminus, using DNA substrates in which the 5′ end-labeled damaged strand contained either a uracil residue or a natural AP site (Figure 3a). When the repair reaction was carried out in the presence of Mg^2+^, a major 28-nt fragment was detected (Figure 3a, lanes 1 and 2), accompanied by smaller fragments probably arising by exonucleolytic degradation. This fragment corresponds to a 3′-OH end, and is probably generated by AP endonuclease incision at the 5′ side of the AP site. When the DNA substrates were incubated with the cell extract in the absence of Mg^2+^, two major fragments appeared (Figure 3a, lanes 5 and 6). These two products co-migrated with those generated on an AP site-containing substrate by the β- and β,δ-elimination activities of the *E. coli* DNA glycosylases/lyases endonuclease III (Nth) and Fpg, respectively (Figure 3a, lanes 3 and 4). Quantification of the relative fluorescence intensity revealed that fragments corresponding to δ-elimination products are threefold more abundant than those representing β-elimination ends (Figure 3a, lanes 5 and 6).

We next analyzed the nature of the 5′ terminus, using DNA substrates in which the damaged strand was labeled at the 3′ end (Figure 3b). As the 5′-blocked ends arising by AP endonuclease activity are known to be alkali labile, the reaction products were stabilized by reduction with sodium borohydride (NaBH_4_) prior to PAGE analysis (Dianov *et al.*, 1992). We found that in the absence of Mg^2+^, the extract catalyzed incision at both the U- and AP-containing substrates to generate a single 22-nt fragment (Figure 3b, lanes 5 and 6). This fragment co-migrated with those produced by *E. coli* Nth or Fpg DNA glycosylases/lyases on an AP site-containing substrate (Figure 3b, lanes 1 and 2), and with the product generated by the dRP-lyase activity of Pol β (Figure 3b, lane 9). Therefore, it probably represents a 5′-P terminus. In contrast, when the reaction was performed in the presence of Mg^2+^ a slightly longer fragment appeared (Figure 3b, lanes 3 and 4). This co-migrated with the 5′-dRP product generated by incubation of the AP:G pair with human AP endonuclease APE1 (Figure 3b, lane 8), and was undetectable when the reaction products were not stabilized with NaBH_4_ (data not shown). Therefore, it probably represents a 5′-dRP blocked terminus.

Taken together, these results indicate that AP sites generated after uracil excision by Arabidopsis cell extracts may be processed either by AP endonucleases or by AP lyases, yielding either 5′- or 3′-blocked ends, respectively.

### Uracil repair involves single-nucleotide insertion and/or long-patch DNA synthesis

As shown in Figure 1b, excision repair of uracil depends on the presence of dNTPs in the reaction mixture, but can also be completed when only dCTP is present. These results suggest that uracil excision repair in Arabidopsis extracts takes place through short- and long-patch DNA synthesis. To explore this possibility, we analyzed the DNA synthesis intermediates arising during the repair reaction.

**Figure 4:**
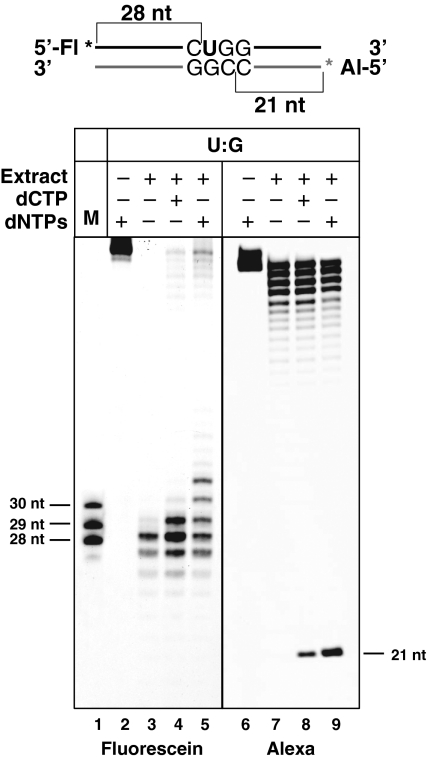
Uracil excision repair involves single-nucleotide insertion and long-patch DNA synthesis. Arabidopsis cell extract was incubated with duplex DNA that contained a U residue in the upper strand, and which was labeled at the 5′ end of the upper strand with fluorescein and at the 5′ end of the lower strand with Alexa Fluor. Reactions were incubated at 30°C for 3 h in a reaction mixture containing either dCTP or all four dNTPs, as indicated. Reaction products were separated in a 12% denaturing polyacrylamide gel either before (lanes 2–5) or after (lanes 6–9) *Hpa*II digestion. Fluorescein- (lanes 2–5) and Alexa Fluor-labeled fragments (lanes 6–9) were detected by fluorescence scanning.

Repair reactions were performed incubating Arabidopsis extracts with a duplex oligonucleotide labeled with fluorescein at the 5′ end of the upper strand, and with Alexa Fluor at the 5′ end of the lower strand (Figure 4). We found that fully repaired products were undetectable in the absence of dNTPs (Figure 4, lane 7), although most of the upper strand was processed to generate a 28-nt fragment corresponding to a free 3′-OH terminus (Figure 4, lane 3). In the presence of dCTP as the only deoxynucleotide, a fully repaired product was detected (Figure 4, lane 8), with the concomitant appearance of a 29-nt fragment on the upper strand, corresponding to the insertion of dCMP in the repair gap (Figure 4, lane 4). Incubation in the presence of all four dNTPs somewhat increased the quantity of fully repaired product (Figure 4, lane 9), and this was accompanied by the accumulation of upper-strand reaction intermediates, which comigrated with 29-, 30- and 31-nt fragments containing free 3′-OH ends (Figure 4, lane 5). These results indicate that up to three nucleotides are inserted when all four dNTPs are present in the repair reaction.

**Figure 5:**
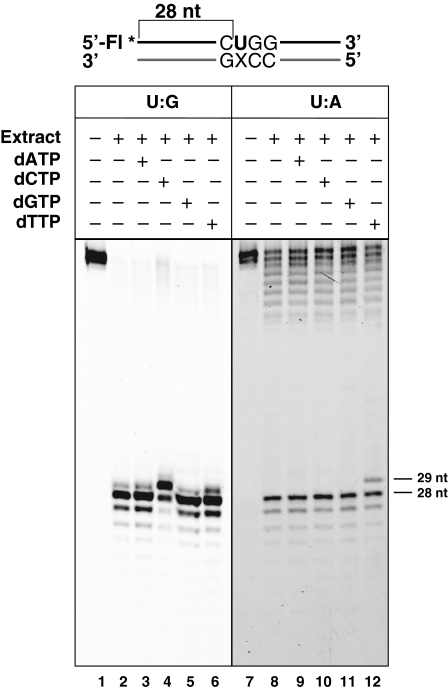
Single-nucleotide insertion after uracil excision is template-dependent. Duplex DNA containing a U residue at the 5′ end-labeled upper strand paired to G (left panel) or A (right panel) was incubated at 30°C for 3 h with Arabidopsis cell extract and a single dNTP, as indicated. Reaction products were separated in a 12% denaturing polyacrylamide gel, and then detected by fluorescence scanning. Fragments corresponding to 28 and 29 nt are indicated by arrows.

We then investigated whether the single-nucleotide insertion observed in the presence of dCTP was template-dependent. We performed repair reactions with DNA substrates containing either a U:G or a U:A pair, in the presence of a single deoxynucleotide (dATP, dCTP, dGTP or dGTP) (Figure 5). Only dCMP or dTMP was inserted in the DNA substrates containing either U:G or U:A, respectively (Figure 5, lanes 4 and 12). The faint bands just above the 28-nt position (Figure 5, lanes 4 and 12) might represent some β-elimination product(s) generated by AP lyases. As previously reported for mammalian cells (Visnes *et al.*, 2008), we found that Arabidopsis extracts processed U:G pairs more efficiently than U:A pairs.

**Figure 6:**
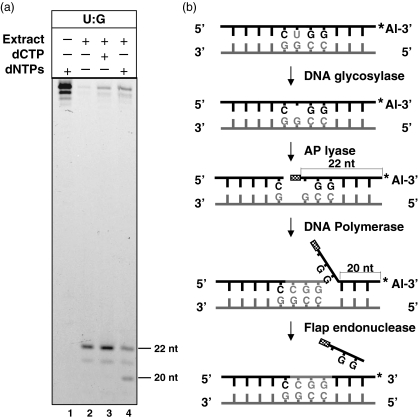
Strand displacement and incision during long-patch DNA synthesis. (a) Duplex DNA containing a U residue at the 3′ end-labeled upper strand was incubated at 30°C for 3 h with Arabidopsis cell extract in a reaction mixture containing either dCTP or all four dNTPs, as indicated. Relevant sizes in nucleotides are shown on the right. (b) Schematic diagram of strand displacement and incision during long-patch BER.

The standard model of LP mammalian BER proposes that, after base excision and AP site incision, the damaged strand undergoes displacement 3′ to the nick, and that the resulting flap structure is finally excised by FEN1 before DNA ligation can occur (Klungland and Lindahl, 1997; Prasad *et al.*, 2000). We therefore looked for evidence of displacement and incision 3′ to the damaged base on the uracil-containing strand when all four dNTPs were present in the reaction mixture. To this end, we performed the repair reaction using a duplex oligonucleotide containing a U:G pair, labeled at the 3′ end of the uracil-containing strand, as the DNA substrate (Figure 6). As expected, irrespective of the presence or absence of dNTPs, a major 22-nt 3′-labeled fragment was generated, which corresponds to the AP incision intermediate (Figure 6a, lane 2). This major intermediate was accompanied by a minor 21-nt fragment that might represent exonucleolytic degradation. However, incubation of the extract and the repair substrate in the presence of all four dNTPs specifically gave rise to a 20-nt 3′-labeled fragment (Figure 6a, lane 4), which is consistent with a 3-nt strand displacement followed by flap excision (Figure 6b).

Taken together, these results suggest that excision repair of uracil by Arabidopsis whole-cell extracts may arise by single-nucleotide insertion, and by an extended (at least 3-nt-long) DNA synthesis involving strand displacement and flap excision.

### DNA repair synthesis is aphidicolin resistant and ddCTP sensitive

**Figure 7:**
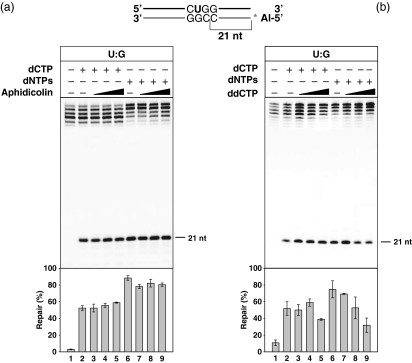
Effects of DNA polymerase inhibitors on uracil excision repair by Arabidopsis cell extracts. Duplex DNA containing U opposite G at a *Hpa*II site was incubated at 30°C for 3 h with Arabidopsis cell extract in a reaction mixture containing either dCTP or all four dNTPs, as indicated, and increasing levels of aphidicolin (left panel: 0, 300, 600 or 1200 μm) or 2′,3′-dideoxycytidine 5′-triphosphate (ddCTP, right panel: 0, 20, 200 or 2000 μm). Reaction products were digested with *Hpa*II, separated in a 12% denaturing polyacrylamide gel and quantified by fluorescence scanning. The percentage of fully repaired DNA product is shown. Values are means with standard errors from two independent experiments.

In mammalian cells, it has been proposed that SP BER is Pol β-mediated, whereas LP BER is dependent of replicative DNA polymerases δ and/or ε (Fortini and Dogliotti, 2007). This is supported by reconstitution experiments with purified enzymes, and also by evidence obtained using specific inhibitors to estimate the relative contribution of both types of enzymes to each BER subpathway (Parlanti *et al.*, 2002). In an initial examination of the requirement of DNA polymerase(s) for the gap-filling step during plant BER, we measured the repair activity of Arabidopsis extracts in the presence of two DNA polymerase inhibitors: aphidicolin and 2′,3′-dideoxycytidine 5′-triphosphate (ddCTP) (Figure 7). Aphidicolin is a tetracyclic diterpenoid that specifically interferes with eukaryotic replicative DNA polymerases (α, δ and ε) (Weiser *et al.*, 1991), and is commonly used to synchronize plant cells in culture (Menges and Murray, 2002). ddCTP is an inhibitor of Pol β-like polymerases (Garcia-Diaz *et al.*, 2002; Singhal *et al.*, 1995).

Aphidicolin exerted no noticeable effect on the level of fully repaired *Hpa*II-sensitive product; neither in the presence of dCTP nor in the presence of all four dNTPs (Figure 7a, lanes 2–9). In contrast, ddCTP caused a significant decrease in the level of full-repair product when reactions were carried out in the presence of all four dNTPS (Figure 7b, lanes 6–9). In repair reactions performed with dCTP as the only deoxynucleotide source, an inhibitory effect was observed at a 100:1 molar ratio of ddCTP:dCTP (Figure 7b, lanes 2–5). We therefore concluded that DNA synthesis after uracil excision is aphidicolin resistant and ddCTP sensitive.

### Partial stimulation of uracil excision repair by addition of exogenous proteins

**Figure 8:**
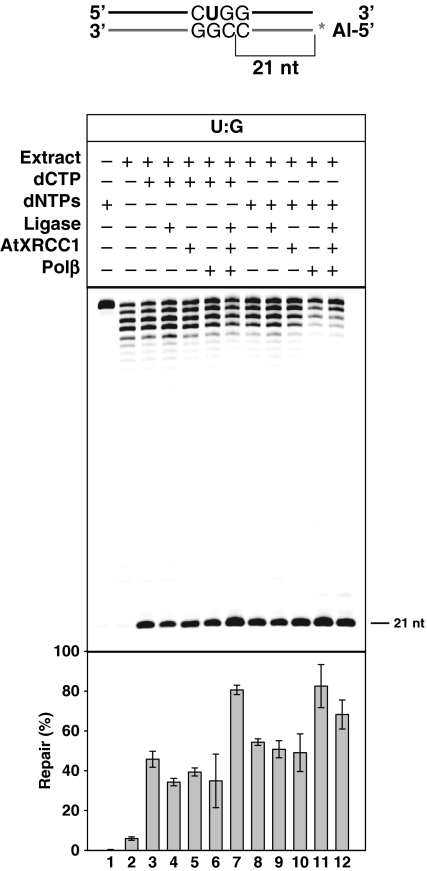
Partial stimulation of uracil repair by the addition of exogenous proteins. Duplex DNA containing U opposite G at a *Hpa*II site was incubated at 30°C for 3 h with Arabidopsis cell extract in a reaction mixture containing either deoxycytidine triphosphate (dCTP) or all four deoxyribonucleotide triphosphates (dNTPs), as indicated, and different combinations of T4 DNA ligase (1.5 U), Arabidopsis AtXRCC1 (65 nm) and human Pol β (2.4 U). Reaction products were digested with *Hpa*II, separated in a 12% denaturing polyacrylamide gel and quantified by fluorescence scanning. The percentage of fully repaired DNA product is shown. Values are means with standard errors from two independent experiments.

As shown in Figure 4, some DNA repair intermediates accumulate during the reaction catalyzed by cell extracts. This may result from low level and/or partial inhibition of some of the protein factors required to complete repair. To explore this possibility, we tested whether the level of fully repaired product might be increased by adding exogenous proteins to the repair reaction. The repair intermediates detected during the reaction included both AP incision and nucleotide insertion products, implying that the DNA synthesis and ligation steps might be rate-limiting. We therefore supplemented the reaction mixture with T4 DNA ligase, human DNA polymerase β or the Arabidopsis ortholog of the mammalian BER scaffold protein XRCC1, as well as with all three proteins together (Figure 8). The concentrations of DNA polymerase and DNA ligase used were in excess over those required to process all DNA substrate (DCC, TMR, TRA and RRA, unpublished data). We found that the simultaneous addition of all three exogenous proteins significantly increased the quantity of fully repaired product in reactions performed with dCTP as the only deoxynucleotide source (Figure 8, lane 7). In reactions with all four dNTPs, addition of Pol β significantly enhanced full repair (Figure 8, lane 11), and some increase was also observed when all three exogenous proteins were added (Figure 8, lane 12). Notwithstanding the fact that the added ligase and DNA polymerase are heterologous proteins, these results suggest that optimal DNA repair *in vitro* by Arabidopsis cell extracts may depend on an appropriate balance of different enzymatic activities and accessory factors.

## Discussion

### An experimental system that specifically detects BER in plants

Previous reports have described DNA repair activities in plant cell extracts, but to our knowledge there is no published description of an experimental *in vitro* system allowing the specific analysis of the BER pathway in plants. A reliable assay has been developed to detect nucleotide excision repair (NER) in Arabidopsis whole-cell extracts, which measures DNA repair synthesis in plasmid substrates treated with UV-C, methylene blue or cisplatin, but does not allow the unambiguous distinction of the relative contributions of NER and BER, especially for oxidative lesions induced by methylene blue (Li *et al.*, 2002). A recent report has described the incision of osmium tetroxide-treated plasmids in chloroplast extracts by DNA glycosylase/lyase and/or by AP endonuclease activities (Gutman and Niyogi, 2009), but not full BER. A previous investigation of BER using rice extracts (Sarkar *et al.*, 2004) did not allow the discrimination between unrepaired substrate and fully-repaired product, as both were detected as a 51-mer labeled fragment.

A major aim of this work was to extend the biochemical analysis of BER to plants by developing an assay for the specific detection of this important repair pathway in Arabidopsis. We used similar cell extracts to those of previous studies (Li *et al.*, 2002; Qi *et al.*, 2005) in conjunction with defined DNA substrates containing uracil, a lesion known to be repaired by base excision in prokaryotes and eukaryotes. The location of the target lesion at an appropriate restriction site allowed detection of fully repaired products, as previously described in mammalian cell extracts (Dianov *et al.*, 1992; Singhal *et al.*, 1995). Using this *in vitro* system we have demonstrated that Arabidopsis whole-cell extracts promote uracil excision repair to convert a U:G mismatch to a normal C:G pair. The initiation of uracil repair, but not the AP incision and gap-filling steps, is entirely dependent on enzymatic activity that is blocked by a specific inhibitor of UDGs of the UNG family. The Arabidopsis genome encodes only a putative member of this protein family (At3G18630), but its role in uracil repair is still unknown. Therefore, the assay system described here allows reliable monitoring of uracil-initiated BER in Arabidopsis cells.

### Processing of AP sites by AP endonucleases and AP lyases

We have used the *in vitro* BER assay in Arabidopsis extracts to initiate the functional characterization of this repair pathway in plants. In the classical model of BER, repair of an AP site proceeds through hydrolysis of the phosphodiester bond 5′ to the abasic site by an AP endonuclease, followed by action of a DNA polymerase that simultaneously removes the 5′-dRP blocking residue, and performs gap filling (Dianov *et al.*, 1992; Singhal *et al.*, 1995). However, bifunctional DNA glycosylases with an intrinsic AP lyase can also cleave the phosphodiester bond 3′ to the AP site, and the subsequent 3′-blocked end is processed by an AP endonuclease, thus generating a gap flanked by conventional 3′-OH and 5′-P ends (Levin and Demple, 1990). The biological significance of the AP lyase activity of bifunctional DNA glycosylases during BER is still unclear, as AP endonuclease is still needed to clear the 3′-blocked end, even after AP processing by AP lyase activity. However, there is some evidence that AP lyases may play an important role during BER in some species. In *Schizosaccharomyces pombe*, for example, genetic and biochemical evidence points to the activity of DNA glycosylase/lyase Nth1 as being mainly responsible for incision at AP sites, which are then processed by the AP endonuclease Apn2 and repaired though an SP subpathway (Alseth *et al.*, 2004).

By performing repair reactions in the presence or absence of Mg^2+^, we have found that AP sites generated after uracil excision may be processed either by AP endonucleases or by AP lyases, generating either 5′- or 3′-blocked ends, respectively. Interestingly, most of the 3′-termini products generated in the absence of Mg^2+^ represented 3′-P ends. This suggests that the AP lyase activity (or activities) involved catalyze β,δ-elimination. This avoids the need for an AP endonuclease to clear the 3′-blocked end, but implies that 3′-phosphoesterase activity may be required to restore conventional 3′-OH. A possible candidate for this role in Arabidopsis is a previously described plant 3′-phosphoesterase that specifically binds to single-strand DNA breaks (Petrucco *et al.*, 2002). In any case, our results are compatible with the hypothesis that AP endonuclease-dependent and -independent BER pathways coexist in plant cells, as reported in mammalian cells (Wiederhold *et al.*, 2004).

At this point it is difficult to assign candidate Arabidopsis genes for the AP endonuclease and AP lyase activities detected in cell extracts. The Arabidopsis genome encodes three putative AP endonucleases (AtAPE1, AtAP2 and AtARP), and only the latter has been shown to perform incisions in DNA containing abasic sites (Babiychuk *et al.*, 1994). On the other hand, there are at least nine Arabidopsis genes encoding bifunctional DNA glycosylases, and for seven of these (AtMMH, AtNTH1, AtOGG1, DME, ROS1, DML2 and DML3), an AP lyase activity has been demonstrated *in vitro* (Agius *et al.*, 2006; Garcia-Ortiz *et al.*, 2001; Gehring *et al.*, 2006; Morales-Ruiz *et al.*, 2006; Ohtsubo *et al.*, 1998; Ortega-Galisteo *et al.*, 2008; Roldan-Arjona *et al.*, 2000).

### Single-nucleotide and long-patch base excision repair of uracil and abasic sites in DNA by Arabidopsis cell extracts

In mammalian cells, processing of AP sites generated after excision is carried out either by single-nucleotide replacement or by long-patch DNA synthesis (Fortini and Dogliotti, 2007). Given the absence of plant homologs of DNA ligase III and DNA polymerase β, which are both required for mammalian SP BER (Kubota *et al.*, 1996), it has recently been proposed that plants only use an LP BER pathway (Uchiyama *et al.*, 2008). However, we have found that BER of uracil and abasic sites in Arabidopsis cell extracts occurs by both single-nucleotide insertion and long-patch DNA synthesis. Three main observations indicate that repair of a U:G mispair may take place via single-nucleotide DNA synthesis: (i) dCTP alone is sufficient to support full repair; (ii) only a 29-nt intermediate is detected in single-nucleotide reactions; and (iii) the only nucleotide inserted is complementary to the base opposite the U residue. On the other hand, we also found evidence of an LP BER when the reaction mixtures contained all four dNTPs. In this case, the reaction intermediates detected included not only 29-, but also 30- and 31-nt 5′-labeled fragments. Importantly, a shortened 3′-labeled fragment was detected in these conditions, consistent with strand displacement DNA synthesis followed by the removal of the DNA flap. Homologs of the mammalian flap endonuclease FEN1 are encoded in the genomes of Arabidopsis and *Oryza sativa* (Kimura and Sakaguchi, 2006), but their role in BER is not known.

In mammalian cells, the factors that determine the selection of either the SP or LP BER pathway are still poorly understood, although it has been proposed that both the nature of the DNA glycosylase and the lesion may influence the choice. Thus, repair of hypoxanthine or ethenoadenine initiated by the ANPG glycosylase is completed via both SP and LP BER (Fortini *et al.*, 1999), whereas excision of 8-oxoG by OGG1 is preferentially followed by an SP BER (Dianov *et al.*, 1998; Fortini *et al.*, 1999). There is evidence that uracil-initiated BER in mammalian cell-free extracts involves SP in about 20% of repair events (Bennett *et al.*, 2001), but the balance between SP and LP may also be modulated by the cell-cycle phase (Fortini and Dogliotti, 2007). Reports on mammalian mitochondrial extracts have variously suggested that uracil excision is followed only by SP BER (Stierum *et al.*, 1999) or by SP and LP BER pathways (Akbari *et al.*, 2008). Findings from non-mammalian systems suggest that the relative relevance of SP and LP pathways in the same lesion may differ, even between related species. Thus, in *Saccharomyces cerevisiae* most AP sites are repaired through LP BER (Boiteux and Guillet, 2004), whereas in *Schizosaccharomyces pombe* genetic and biochemical evidence points to the preeminence of the SP pathway (Alseth *et al.*, 2004). We found that the kinetics of BER by Arabidopsis extracts is faster when all dNTPs are present in the repair reaction, but that the rate of SP BER is nonetheless also quite significant. It will be important to determine whether these two pathways occur in competition in plant cells, and to identify the factors determining their relative significance *in vivo*. Resolving these and other questions will require experimental data on different DNA lesions, and the identification of enzymes specifically involved either in SP or LP BER.

### The nature of DNA polymerases involved in plant BER

We have explored the nature of the DNA polymerase(s) involved in the repair synthesis step during plant BER by performing repair assays in the presence of two inhibitors. Our results indicate that DNA synthesis after uracil excision is aphidicolin resistant and ddCTP sensitive. In contrast, the DNA synthesis observed during NER in Arabidopsis cell extracts is both aphidicolin- and ddTTP-resistant (Li *et al.*, 2002). Uracil-initiated BER in mammalian cells is completely inhibited by ddCTP and unaffected by aphidicolin, thus supporting the idea that DNA polymerase β is required for the gap-filling step (Singhal *et al.*, 1995). However, the BER of AP sites in mammalian cells is partially aphidicolin sensitive, and the analysis of Pol β-proficient and -deficient cells has led to the proposal that there is a competition between distributive and processive DNA polymerases for nucleotide addition at the primer terminus (Parlanti *et al.*, 2004). The Arabidopsis genome does not encode any ortholog of DNA polymerase β, but contains a gene for DNA polymerase λ (Garcia-Diaz *et al.*, 2000), another member of the X DNA polymerase family that also contributes to BER in mammalian cells (Braithwaite *et al.*, 2005). Pol λ is the only X family-DNA polymerase identified in plants (Uchiyama *et al.*, 2008), and *O. sativa* Pol λ is ddTTP sensitive and aphidicolin resistant (Uchiyama *et al.*, 2004), like its mammalian counterpart (Garcia-Diaz *et al.*, 2002; Shimazaki *et al.*, 2002).

Therefore, our results are consistent with a possible role of Arabidopsis Pol λ during uracil-initiated BER. However, it should be noted that inhibition by ddCTP was less intense under conditions of single-nucleotide insertion, and that substantial DNA repair persisted, with both SP and LP, even at a 100:1 molar ratio of ddCTP:dCTP. Therefore, it is possible that, as in mammalian cells, there is competition between different DNA polymerases for access to the repair gap. Nevertheless, the information available about plant DNA polymerases and their role in repair is still too limited to suggest tentative candidates for DNA synthesis during plant BER.

### Control of BER rate and accumulation of DNA repair intermediates

BER is a multistep process, and it is likely that the individual enzymatic activities involved in this repair pathway are coordinated *in vivo* to maximize repair efficiency and minimize the persistence of deleterious repair intermediates. Not unexpectedly, this coordination is to some extent lost when BER is recapitulated *in vitro*. We have found that the rate of uracil repair by Arabidopsis whole-cell extracts reaches a plateau after about 3 h, and not all DNA substrates are fully repaired even after longer incubation times (data not shown). As this is accompanied by the accumulation of AP incision and nucleotide insertion products, it is likely that the rate-limiting steps are neither uracil excision nor AP processing, but rather some of the factors required during gap filling and/or ligation. In fact, we found that the exogenous addition of heterologous DNA ligase and DNA polymerase β activities, as well as of the Arabidopsis scaffold protein AtXRCC1, to some extent increase the level of fully repaired product. However, it is also conceivable that the repair rate is additionally controlled by some inhibitory factor(s) in the extracts. In mammalian cells, the poly(ADP-ribose) polymerase PARP-1 has a high affinity for the single-strand-break intermediates generated during BER (Lindahl *et al.*, 1995), and has been reported to have a negative effect on BER repair rates in cell extracts (Allinson *et al.*, 2003, 2004). The Arabidopsis genome encodes two PARP proteins that are induced by DNA damage (Babiychuk *et al.*, 1998; Doucet-Chabeaud *et al.*, 2001), but it is not known whether there is any PARP activity in the Arabidopsis extracts used in our study. In any case, we envisage that the experimental system described here may be useful for identifying important regulatory factors that control the rate of BER in plant cells.

## Experimental procedures

### Plant material

Sterilized seeds of wild-type *Arabidopsis thaliana* plants (ecotype Columbia) were plated on 10-cm-diameter Petri dishes containing 25 ml of 0.44% (w/v) MS medium (Sigma-Aldrich, http://www.sigmaaldrich.com), supplemented with 3% (w/v) sucrose and 0.8% (w/v) agar, pH 5.8. Plates were transferred to the growth chamber under long-day conditions (16-h light/8-h dark) at 23°C. After 15 days, whole plants were harvested, frozen in liquid nitrogen and stored at −80°C.

### Plant cell extract preparation

Seedling extracts were prepared by introducing several modifications to previously published methods (Li *et al.*, 2002; Qi *et al.*, 2005). All steps were performed at 0–4°C. Frozen plant material was ground in a handle mortar with liquid N_2_, and the resulting powder was resuspended in 2–3 volumes (w/v) of homogenization buffer containing 25 mm HEPES-KOH, pH 7.8, 100 mm KCl, 5 mm MgCl_2_, 250 mm sucrose, 10% glycerol, 1 mm DTT and 1 μl ml^−1^ protease inhibitor cocktail (Sigma-Aldrich). The homogenate was incubated for 30 min at 4°C and centrifuged at 13 000 *g* for 1 h. The supernatant was filtered through a 20-μm nylon mesh and dialyzed overnight against 25 mm HEPES-KOH, pH 7.8, 100 mm KCl, 17% glycerol and 2 mm DTT. Protein concentration was determined by the Bradford assay with BSA standards, and the extract was stored in small aliquots at −80°C.

### Reagents and enzymes

*Escherichia coli* UDG, human AP endonuclease APE1 and Ugi were obtained from New England BioLabs (http://www.neb.com). Human DNA polymerase β was from Trevigen (http://www.trevigen.com). *E. coli* T4 DNA ligase was purchased from Promega (http://www.promega.com) and *Hpa*II was purchased from Roche (http://www.roche-applied-science.com). *E. coli* Nth and Fpg DNA glycosylases/lyases were prepared as previously described (Roldan-Arjona *et al.*, 1996, 1997). Partially purified AtXRCC1 from *A. thaliana* was a kind gift from M.I. Martínez-Macías (University of Córdoba, Spain).

### DNA substrates

Oligonucleotides used to prepare DNA substrates (Table S1) were synthesized by Operon (http://www.operon-biotech.com), and were purified by PAGE before use. Double-stranded DNA substrates were prepared by mixing a 5-μm solution of the upper-strand oligonucleotide (labeled at the 5′ or 3′ end with fluorescein, where indicated) with a 10-μm solution of the lower-strand oligonucleotide (labeled at the 5′ end with Alexa Fluor 647, where indicated), heating to 95°C for 5 min and slowly cooling to room temperature (25°C). DNA containing a natural AP site opposite guanine was prepared by incubating a DNA duplex containing a U:G mispair, prepared as above, with *E. coli* UDG (0.5 U) at 30°C for 5 min.

### DNA repair reactions in seedling extracts

Repair reactions (50 μl) contained 45 mm HEPES-KOH, pH 7.8, 70 mm KCl, 5 mm MgCl_2_, 1 mm DTT, 0.4 mm EDTA, 2 mm ATP, 36 μg BSA, 1 mm NAD, 2% glycerol, 20 μm each deoxynucleotide (dCTP, dGTP, dATP and dTTP, except where indicated), substrate DNA (40 nm) and 70 μg of extract. After incubation at 30°C for 3 h, reactions were stopped by adding 20 mm EDTA, 0.6% SDS and 0.5 mg ml^−1^ proteinase K, and the mixtures were incubated at 37°C for 30 min. DNA was extracted with phenol:chloroform:isoamyl alcohol (25:24:1) and ethanol-precipitated at −20°C in the presence of 0.3 mm NaCl and 16 μg ml^−1^ glycogen. Samples were resuspended in 10 μl of 90% formamide, and were heated at 95°C for 5 min. Where indicated, DNA was resuspended in 5 μl of SuRE/Cut Buffer L containing 5 U of *Hpa*II (Roche) and incubated at 37°C for 1 h. Reactions were then stopped by adding 5 μl of 90% formamide and then heating at 95°C for 5 min. Reaction products were separated in a 12% denaturing polyacrylamide sequencing gel (40 × 20 cm) containing 7 m urea. Fluorescein- or Alexa-labeled DNA was visualized in an FLA-5100 imager, and data were analyzed using Multigauge (Fujifilm, http://www.fujifilm.com).

### Analysis of 5′ and 3′ ends generated during AP site processing

Repair reactions (50 μl) were performed at 30°C for 3 h in the buffer indicated above (with no dNTPs), either in the presence or absence of 5 mm MgCl_2_. Control AP lyase reactions were performed by incubating a DNA containing a natural AP site opposite guanine with *E. coli* Nth or Fpg at 37°C, for 30 or 60 min, respectively. Control AP endonuclease reactions were performed by incubating a DNA containing a natural AP site opposite guanine with human APE1 (10 U) at 37°C for 60 min, supplemented with 2.4 U of human DNA polymerase β when generating a control dRP-lyase product. When analyzing 5′ ends, reaction products were stabilized by the addition of freshly prepared sodium borohydride (NaBH_4_; Sigma-Aldrich) to a final concentration of 300 mm, incubation at 0°C for 30 min and desalting in a microspin G-25 column (GE Healthcare, http://www.gelifesciences.com). All reaction products were purified, separated and analyzed as described above.

## SUPPORTING INFORMATION

**Figure S1.** DNA ends generated after AP site cleavage by AP endonucleases and AP lyases.

**Table S1.** DNA sequences of the oligonucleotides used as substrates.
