# Supplementary material for: Single-nucleotide and long-patch base excision repair of DNA damage in plants
Source: Plant J. 2009 Sep 1;60(4):716–28. doi: 10.1111/j.1365-313X.2009.03994.x (PMC2954439; doi:10.1111/j.1365-313X.2009.03994.x)
Supplement: Supplementary file 1 [file tpj0060-0716-SD1.pdf]

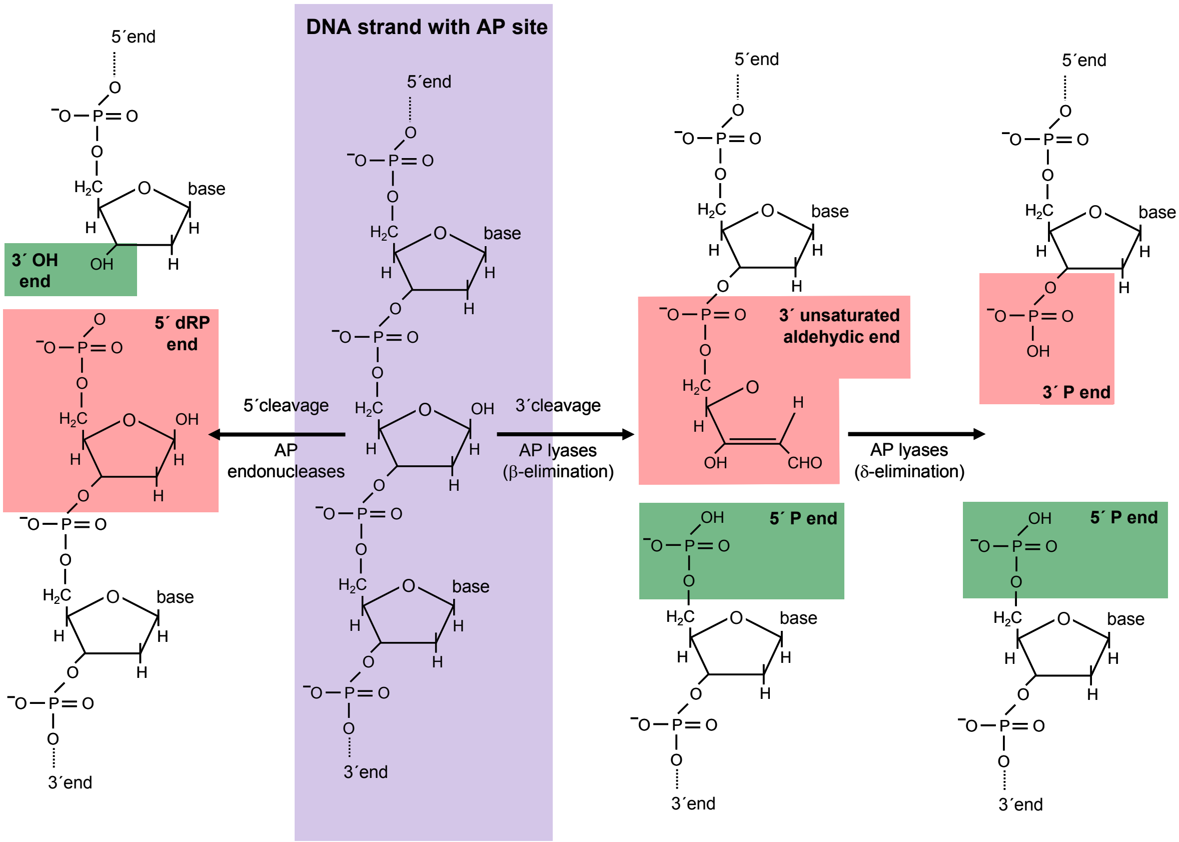

Figure S1. DNA ends generated after AP site cleavage by AP endonucleases and AP lyases. Cleavage by AP endonucleases takes place at the 5´side of the AP site and yields a 3´-OH end and a 5´-deoxyribose-5-phosphate (5´-dRP) terminus. By contrast AP lysases cleave 3´ to the AP site through a β-elimination mechanism, leaving a 5´-phosphate end and a 3´-α,β-unsaturated aldehyde; in some cases, they additionally perform a δ-elimination event that generates a 3´-phosphate end [Adapted from: Boiteux, S. and Guillet, M. (2004) Abasic sites in DNA: repair and biological consequences in *Saccharomyces cerevisiae*. *DNA Repair (Amst)*, 3, 1-12.]
