# Supplementary material for: Single-nucleotide and long-patch base excision repair of DNA damage in plants
Source: Plant J. 2009 Sep 1;60(4):716–28. doi: 10.1111/j.1365-313X.2009.03994.x (PMC2954439; doi:10.1111/j.1365-313X.2009.03994.x)
Supplement: Supplementary file 2 [file tpj0060-0716-SD2.pdf]

Table S1. DNA sequence of oligonucleotides used as substrates.

Name	DNA sequence[a]	Strand	Label[b]
Fl-UGF	TCACGGGATCAATGTGTTCTTTCAGCTC**U**GGTCACGCTGACCAGGAATACC	Upper	Fl at 5'
UGF-Fl	TCACGGGATCAATGTGTTCTTTCAGCTC**U**GGTCACGCTGACCAGGAATACC	Upper	Fl at 3'
Fl-APGF	TCACGGGATCAATGTGTTCTTTCAGCTC**F**GGTCACGCTGACCAGGAATACC	Upper	Fl at 5'
CGR	AGTGCCCTAGTTACACAAGAAAGTCGAG**G**CCAGTGCGACTGGTCCTTATGG	Lower	-
Al-CGR	AGTGCCCTAGTTACACAAGAAAGTCGAG**G**CCAGTGCGACTGGTCCTTATGG	Lower	Al at 5'

[a]F = AP site analog (tetrahydrofuran)

[b]Fl = fluorescein; Al = alexa fluor 647
